# Association between perioperative non-steroidal anti-inflammatory drug use and cardiovascular complications after non-cardiac surgery in older adult patients

**DOI:** 10.1186/s40981-024-00712-5

**Published:** 2024-04-30

**Authors:** Ryu Komatsu, Michael D. Singleton, Emily M. Dinges, Jiang Wu, Laurent A. Bollag

**Affiliations:** 1 Department of General Anesthesiology and Department of Outcomes Research, Anesthesiology Institute, Cleveland Clinic, Cleveland, OH USA; 2 Institute of Translational Health Sciences, University of Washington, Seattle, WA USA; 3 Department of Anesthesiology and Pain Medicine, University of Washington, Seattle, WA USA

**Keywords:** NSAIDs, Elderly, Cardiovascular complication, Renal complication, Gastrointestinal complication

## Abstract

**Background:**

We investigated in older adult non-cardiac surgical patients whether receipt of perioperative non-steroidal anti-inflammatory drugs (NSAIDs) is associated with increased incidence of postoperative cardiovascular complications.

**Methods:**

We retrospectively extracted the information for patients with age ≥  65 years who had inpatient non-cardiac surgery with a duration of ≥  1 h from the American College of Surgeons-National Surgical Quality Improvement Program registry data acquired at the University of Washington Medical Center. We compared patients who received NSAIDs perioperatively to those who did not receive NSAIDs, on the two composite outcomes: (1) the incidence of postoperative cardiovascular complications within 30 days of the surgery, and (2) the incidence of combined postoperative gastrointestinal and renal complications, and length of postoperative hospital stay. We used separate multivariable logistic regression models to analyze the two composite outcomes and a Poisson regression model for the length of hospital stay.

**Results:**

The receipt of perioperative NSAIDs was not associated with postoperative cardiovascular complications (estimated odds ratio (OR), 1.78; 95% confidence interval (CI), 0.97 to 3.25; *P* =  0.06), combined renal and gastrointestinal complications (estimated OR, 1.30; 95% CI, 0.53 to 3.20; *P* =  0.57), and length of postoperative hospital stay in days (incidence rate ratio, 1.06; 95% CI, 0.93 to 1.21; *P* =  0.39).

**Conclusions:**

In older adult non-cardiac surgical patients, receipt of perioperative NSAIDs was not associated with increased incidences of postoperative cardiovascular complications, and renal and gastrointestinal complications within 30 days after surgery, or length of postoperative hospital stay.

**Supplementary Information:**

The online version contains supplementary material available at 10.1186/s40981-024-00712-5.

## Background

Non-steroidal anti-inflammatory drugs (NSAIDs) effectively reduce inflammation, relieve pain, and have been used to treat a variety of conditions such as rheumatoid arthritis, osteoarthritis, gout, and back aches. Perioperative use of NSAIDs as part of a multimodal analgesic approach has been shown to be beneficial in reducing the use of opioids, and opioid-related adverse effects [1, 2], and potentially hastens postoperative recovery [3, 4].

However, concerns regarding the association between NSAID use and increased risk of cardiovascular thrombotic events have been raised. A systematic review by Hernandez-Diaz et al. has shown an increased risk of MI with rofecoxib, ibuprofen, and diclofenac [5]. Another systematic review by McGettigan et al. has shown an association between increased cardiovascular risks with rofecoxib and diclofenac [6]. Initial analyses of cardiovascular risk with rofecoxib indicated that 7 to 18 months of exposure to rofecoxib were required: however, subsequent analysis indicated increased risk early in treatment and even with the first dose [7, 8].

It has been noted that even a short duration of exposure to non-selective NSAIDs, such as ketorolac and meloxicam in some populations is sufficient to increase cardiovascular risk [9]. Mechanisms for increased cardiovascular risk with cyclooxygenase (Cox)-2 selective inhibitors include prostacyclin-thromboxane imbalance leading to prothrombotic effects of the platelet-endothelium interface. Adverse cardiovascular outcomes caused by non-selective NSAIDs are also considered to be related to thrombotic events. Although non-selective NSAIDs suppress both Cox-1 and Cox-2 enzymes, some preparations have more potent Cox-2 inhibitory activity than Cox-1 inhibitory activity (i.e., diclofenac, piroxicam), and other preparations have more potent Cox-1 inhibitory activity (i.e., ibuprofen, naproxen) [10]. Those with relative Cox-2 selectivity (i.e., diclofenac) have been associated with a higher risk of cardiovascular events than those with less potent Cox-2 activity (i.e., naproxen) [6].

Several studies have suggested increased trends of cardiovascular events in cardiac surgery patients with the use of non-selective NSAIDs [11, 12], but few information has been available regarding a possible link between perioperative NSAIDs use and cardiovascular events after non-cardiac surgery, and the information specifically for older adult patients who are particularly at high risk of postoperative cardiovascular complications are lacking to date.

We therefore tested the primary hypothesis that older adult (age ≥  65 years) non-cardiac surgical patients who received NSAIDs intra- or postoperatively before discharge from the hospital have increased incidence of the composite of cardiovascular complications within 30 days after surgery than those who did not receive NSAIDs. Our secondary hypotheses were that patients who receive NSAIDs intra- or postoperatively have increased incidences of combined composite of renal and gastrointestinal complications, and increased length of postoperative hospital stay than those who did not receive NSAIDs.

## Methods

After institutional review board approval and waiver of consent (University of Washington IRB ID: STUDY00012619, approval date: 2/25/2021), we extracted the information for patients age ≥  65 years who had in-patient non-cardiac surgery with a duration of 1 h or longer between 2011 and 2020 from the American College of Surgeons-National Surgical Quality Improvement Program (ACS-NSQIP) registry data acquired at the University of Washington Medical Center. Patients who had surgeries that lasted less than 1 h, and ambulatory surgeries were excluded. The NSQIP contained variables describing preoperative conditions, perioperative events, and postoperative complications through 30 days. The information that was not available in the NSQIP database was acquired by case-by-case chart review using the patient’s medical record number. This study followed the Strengthening the Reporting of Observational Studies in Epidemiology (STROBE) reporting guideline.

Perioperative exposure to NSAIDs was defined as receipt of NSAIDs intraoperatively or postoperatively during postoperative admission after index surgery (including both Cox-2 selective inhibitors and non-selective NSAIDs).

### Outcomes

The primary outcome was the composite of postoperative cardiovascular complications as defined by NSQIP pulmonary embolism, cerebrovascular accident, cardiac arrest requiring cardiopulmonary resuscitation (CPR), myocardial infarction, or vein thrombosis requiring therapy within 30 days after surgery (https://www.facs.org/quality-programs/data-and-registries/acs-nsqip/participant-use-data-file/, last accessed on 12/19/2023). The secondary outcome was the combined composite of postoperative renal and gastrointestinal complications. Items included in renal complications were progressive renal insufficiency and acute renal failure, both defined by NSQIP. Items included in gastrointestinal complications were acute gastric ulcer without hemorrhage or perforation, gastric ulcer unspecified as acute or chronic without hemorrhage or perforation, acute duodenal ulcer without hemorrhage, acute peptic ulcer site unspecified without hemorrhage, acute gastritis without bleeding, other gastritis without bleeding, other gastritis with bleeding, duodenitis without bleeding, duodenitis with bleeding, hemorrhage of anus and rectum, hematemesis, melena blood in stool, or gastrointestinal hemorrhage unspecified, within 30 days after surgery, as defined by International Classification of Diseases (ICD)-9 or ICD-10 codes made within 30 days after surgery (Supplemental Table S1). The other secondary outcome was the length of postoperative hospital stay in days.

### Statistical analyses

Univariable tests of association between patient characteristics and intraoperative or postoperative treatment with NSAIDs were conducted using Pearson’s chi-squared test for categorical variables, and *t* test or Wilcoxon Mann–Whitney *U* test for continuous variables. Distributions of continuous variables, by treatment group, were assessed visually using histograms.

We used inverse probability of treatment weighting to adjust for observed potential confounding variables. Specifically, we first estimated the probability of receiving NSAIDs (i.e., propensity score) for each patient using logistic regression, with all baseline characteristics in Table 1 as independent variables. Second, we calculated stabilized inverse propensity score weights (i.e., 1 for patients who received NSAID intraoperatively or postoperatively and $\widehat{p}$/(1 − $\widehat{p}$) for patients who did not, where $\widehat{p}$ is the estimated propensity score). Last, we used the stabilized inverse propensity score weights in all regression models to reduce potential confounding when comparing NSAIDs and non-NSAID patients on outcomes and to estimate the average treatment effect for treated patients. We assessed the covariate balance achieved by propensity score weighting by reporting the absolute standardized mean difference (ASMD) between the treatment groups. ASMD is defined as the absolute difference in means, mean ranks, or proportions divided by the pooled standard deviation. We reported both the weighted and unweighted ASMDs. Variables having ASMD >  0.2 after propensity score weighting were defined as imbalanced and were included as covariates in the weighted regression models.

**Table 1:** Demographic, preoperative, and operative characteristics

	NSAID (*n* = 718)	Non-NSAID (*n* = 2167)	*P* value
Age, years—median [IQR]	70.1 [6.2]	71.6 [8.6]	< 0.001
Sex, female	499 (69.5)	1153 (53.2)	< 0.001
Ethnicity			0.46
Native American/Pacific Islander	15 (2.1)	52 (2.4)	
Asian	39 (5.4)	39 (6.4)	
African American	15 (2.1)	68 (3.1)	
White	635 (88.4)	1872 (86.4)	
Unknown	14 (2.0)	36 (1.7)	
BMI, kg/m^2^	27.8 ± 6.4	28.5 ± 7.0	0.03
Current smoker, *N* (%)	58 (8.1)	127 (5.9)	0.04
ASA physical status, *N* (%)			< 0.001
1–2	208 (29.0)	491 (22.7)	
3	482 (67.1)	1,441 (66.5)	
4–5	28 (3.9)	235 (10.8)	
Functional health status prior to current illness, *N* (%)			0.02
Independent	713 (99.3)	2139 (98.7)	
Partially or totally dependent	2 (0.3)	22 (1.2)	
Unknown	3 (0.4)	2 (0.1)	
Ventilator dependent, *N* (%)	0 (0)	13 (0.6)	0.04
History of severe COPD, *N* (%)	28 (3.9)	110 (5.1)	0.20
Ascites within 30 days prior to surgery, *N* (%)	10 (1.4)	23 (1.1)	0.27
Congestive heart failure in 30 days before surgery, *N* (%)	2 (0.3)	28 (1.3)	0.02
Disseminated cancer, *N* (%)	54 (7.5)	152 (7.0)	0.65
Steroid use for chronic condition, *N* (%)	47 (6.6)	236 (10.9)	0.001
Myocardial infarction 6 months prior to surgery, *N* (%)	2 (0.3)	4 (0.2)	0.63
Coronary artery disease, *N* (%)	219 (30.5)	747 (34.5)	0.05
Hypertension requiring medication, *N* (%)	294 (41.0)	1198 (55.3)	< 0.001
Stroke, *N* (%)	19 (2.7)	126 (5.8)	0.001
Transient ischemic attack, *N* (%)	17 (2.4)	90 (4.2)	0.03
Cerebrovascular disease, *N* (%)	23 (3.2)	118 (5.5)	0.02
Bleeding disorders, *N* (%)	26 (3.6)	139 (6.4)	0.005
Acute renal failure within 24 h prior to surgery, *N* (%)	2 (0.3)	16 (0.8)	0.18
Currently on dialysis, *N* (%)	2 (0.3)	25 (1.2)	0.04
Diabetes, *N* (%)			0.002
No	627 (87.3)	1786 (82.4)	
Insulin	33 (4.6)	178 (8.2)	
No insulin	58 (8.1)	203 (9.4)	
Preoperative transfusion > 4 units PRBCs in 72 h before surgery, *N* (%)	4 (0.6)	25 (1.2)	0.17
Sepsis, *N* (%)			0.002
No	707 (98.5)	2082 (96.1)	
Yes	11 (1.5)	85 (3.9)	
Preoperative NSAID use, *N* (%)	161 (22.4)	267 (12.3)	< 0.001
Year of surgery, *N* (%)			< 0.001
2011	14 (1.9	99 (4.6)	
2012	49 (6.8)	272 (12.6)	
2013	55 (7.7)	251 (11.6)	
2014	68 (9.5)	284 (13.1)	
2015	104 (14.5)	238 (11.0)	
2016	112 (15.6)	234 (10.8)	
2017	90 (12.5)	202 (9.3)	
2018	96 (13.4)	232 (10.7)	
2019	79 (11.0)	209 (9.6)	
2020	51 (7.1)	146 (6.7)	
Emergency surgery, *N* (%)	14 (2.0)	147 (6.8)	< 0.001
General anesthesia, *N* (%)	716 (99.7)	2155 (99.4)	0.36
Surgical procedure, *N* (%)			< 0.001
General surgery	460 (64.1)	1883 (86.9)	
Gynecology	254 (35.4)	193 (8.9)	
Thoracic or vascular	4 (0.6)	91 (4.2)	
Duration of surgery, minutes	238 ± 124	239 ± 137	.84

Summary statistics presented as *N* (%) of patients, mean ± SD, or median [IQR]

*Abbreviations: SD *standard deviation, *IQR *interquartile range, *NSAID *non-steroidal anti-inflammatory drug, *BMI *body mass index, *ASA *American Society of Anesthesiologists, *COPD *chronic obstructive pulmonary disease, *PRBC *packed red blood cells, *SIRS *systemic inflammatory response syndrome

The primary outcome was the composite of cardiovascular complications within 30 days after surgery. We assessed the effects of receiving NSAIDs intra- or postoperatively on the composite of cardiovascular complications using a logistic regression model. A subgroup analysis separately investigated the associations of intra- and postoperative exposure to ibuprofen and ketorolac with the primary outcome, using a similar approach.

We investigated the association of intra- or postoperative treatment with NSAIDs with the incidence of a combined composite of renal and gastrointestinal complications and postoperative length of stay in the hospital in a number of days using logistic regression and generalized Poisson regression models, respectively.

Sensitivity analyses were carried out for both the primary and secondary analyses, as an additional check on the possible impact of extreme weights. For sensitivity analyses, observations having estimated weights lower than the 1st percentile or larger than the 99th percentile were excluded from the regression models.

For all analyses, a nominal type I error rate of 5% was used for interpreting statistical significance. Statistical analysis was carried out using Stata Version 17 (StataCorp, College Station, TX, USA).

## Results

### Patient characteristics and preparations of NSAIDs

We retrieved the data of 2885 unique patients aged ≥  65 who had in-patient non-cardiac surgery with a duration of 1 h or longer at the University of Washington Medical Center between 2011 and 2020 from the American College of Surgeons-National Surgical Quality Improvement Program (ACS-NSQIP) data based on the University of Washington Medical Center, of whom 718 received NSAIDs intra- or postoperatively, while 2167 did not receive NSAIDs. Results of univariable tests of association between patient characteristics and intraoperative or postoperative treatment with NSAIDs are shown in Table 1. Specific preparation of NSAIDs and their doses given are described in Table 2. Ibuprofen and ketorolac were the most used, and few patients received indomethacin and celecoxib (Table 2).

**Table 2:** Total dose of postoperative NSAIDs

NSAID preparation	Total postoperative dose	*N*
Ibuprofen (mg)	2400 [1200–4200] [200–15000]	472
Ketorolac (mg)	60 [15–82] [4–555]	377
Indomethacin (mg)	75 [50–100] [50–100]	2
Celecoxib (mg)	200 [200–800] [200–1000]	11

Summary statistics are presented as median [25th percentile–75th percentile][range]

*Abbreviations*: *NSAID* non-steroidal anti-inflammatory drug. No patients received NSAID preparation other than ibuprofen, ketorolac, indomethacin, and celecoxib

### Crude incidences of cardiovascular renal and gastrointestinal complications

Incidences of components of the composite of cardiovascular complications (primary outcome) and combined composite of renal and gastrointestinal complications (secondary outcome) are described in Table 3. Among patients who received NSAIDs, the incidences of cardiovascular, renal, and gastrointestinal complications were 2.5%, 0.1%, and 0.8%, respectively. Those among patients who did not receive NSAIDs were 1.9%, 0.9%, and 0.8%, respectively. The incidence of overall complications was 3.2% and 3.3% for NSAID and non-NSAID patients, respectively.

**Table 3:** Incidences of the composite of postoperative 30-day cardiovascular, renal, and gastrointestinal complications

	NSAID (*n* = 718)	Non-NSAID (*n* = 2167)	*P* value
Cardiovascular complications	18 (2.5)	42 (1.9)	0.36
Pulmonary embolism	6 (0.8)	10 (0.5)	
Cerebrovascular accident	1 (0.1)	7 (0.3)	
Cardiac arrest requiring CPR	5 (0.7)	8 (0.4)	
Myocardial infarction	2 (0.3)	4 (0.2)	
Vein thrombosis requiring therapy	4 (0.6)	14 (0.7)	
Renal complications	1 (0.1)	19 (0.9)	0.04
Progressive renal insufficiency	1 (0.1)	8 (0.4)	
Acute renal failure	0 (0)	11 (0.5)	
Gastrointestinal complications	6 (0.8)	17 (0.8)	0.89
Acute gastric ulcer without hemorrhage or perforation	0 (0)	0 (0)	
Gastric ulcer, unspecified as acute or chronic, without hemorrhage or perforation	0 (0)	0 (0)	
Acute duodenal ulcer without hemorrhage	0 (0)	0 (0)	
Acute peptic ulcer, site unspecified, without hemorrhage	0 (0)	0 (0)	
Acute gastritis without bleeding	0 (0)	0 (0)	
Other gastritis without bleeding	0 (0)	0 (0)	
Other gastritis with bleeding	0 (0)	0 (0)	
Duodenitis without bleeding	0 (0)	0 (0)	
Duodenitis with bleeding	0 (0)	0 (0)	
Hemorrhage of anus and rectum	0 (0)	2 (0.1)	
Hematemesis	0 (0)	0 (0)	
Melena, blood in stool	3 (0.4)	7 (0.3)	
Gastrointestinal hemorrhage, unspecified	3 (0.4)	14 (0.7)	
Composite of renal and gastrointestinal complications	7 (1.0)	35 (1.6)	0.22
Overall complications	23 (3.2)	71 (3.3)	0.92

Summary statistics presented as *N* (%) of patients, and univariate *P* values

*Abbreviations*: *NSAID* non-steroidal anti-inflammatory drug, *CPR* cardiopulmonary resuscitation

### Covariate balance after propensity score weighting

After weighting by stabilized inverse propensity score weights, potential confounding variables were well balanced between NSAID and non-NSAID patients (i.e., ASMD ≤  0.2) (Table 4).

**Table 4:** Absolute standardized mean differences for baseline and preoperative characteristics, before and after propensity score weighting

	Absolute standardized mean difference	
Variable	Unweighted	Weighted
Age, years	− 0.31	− 0.002
Sex	− 0.34	− 0.037
Ethnicity	0.06	0.009
BMI, kg/m^2^	− 0.11	− 0.11
Current smoker	0.09	− 0.021
ASA physical status	− 0.25	− 0.022
Functional health status prior to current illness	− .0.07	0.011
Ventilator dependent	− 0.11	− 0.045
History of severe COPD	− 0.06	− 0.021
Ascites within 30 days prior to surgery	0.03	0.012
Congestive heart failure 30 days before surgery	− 0.12	0.0
Disseminated cancer	0.02	− 0.017
Steroid use for chronic condition	− 0.15	− 0.015
Myocardial infarction 6 months prior to surgery	0.02	0.012
Coronary artery disease	− 0.09	0.006
Hypertension requiring medication	− 0.29	− 0.023
Stroke	− 0.16	− 0.004
Transient ischemic attack	− 0.10	− 0.011
Cerebrovascular disease	− 0.11	− 0.014
Bleeding disorders	− 0.13	0.004
Acute renal failure within 24 h prior to surgery	− 0.07	− 0.003
Currently on dialysis	− 0.10	0.001
Diabetes	− 0.10	− 0.015
Preoperative transfusion > 4 units PRBCs in 72 h before surgery	− 0.07	0.008
Sepsis	− 0.15	0.001
Preoperative NSAID use	0.27	0.015
Year of surgery	0.26	0.027
Emergency surgery	− 0.24	− 0.005
General anesthesia	0.04	− 0.012
Surgical procedure	0.40	0.028
Duration of surgery, min	− 0.01	− 0.04

*Abbreviations*: *BMI* body mass index, *ASA* American Society of Anesthesiologists, *COPD* chronic obstructive pulmonary disease, *PRBC* packed red blood cells, *NSAID* non-steroidal anti-inflammatory drug

### Outcomes after propensity score weighting

The receipt of NSAIDs was not associated with postoperative cardiovascular complications (estimated odds ratio (OR), 1.78; 95% confidence interval (CI), 0.97 to 3.25; *P* =  0.06), combined renal and gastrointestinal complications (estimated OR, 1.30; 95% CI, 0.53 to 3.20; *P* =  0.57), and length of postoperative hospital stay in days (estimated incidence rate ratio, 1.06; 95% CI, 0.93 to 1.21; *P* =  0.39). In a subgroup analysis of patients who received ibuprofen or ketorolac for the primary outcome, neither ibuprofen (estimated OR, 1.68; 95% CI, 0.86 to 3.28; *P* =  0.13), nor ketorolac (estimated OR, 1.31; 95% CI, 0.60 to 2.83; *P* =  0.49) was associated with the postoperative cardiovascular complications.

### Sensitivity analyses

To assess the possible influence of extreme propensity score weights on regression results, we ran all regression models a second time using 1% trimmed propensity score weights. Trimming resulted in the exclusion of 200 patients (7% of the total sample) from the sensitivity analysis. In the sensitivity analyses, the receipt of NSAIDs was not associated with postoperative cardiovascular complications (estimated OR, 1.68; 95% CI, 0.86 to 3.28; *P* =  0.13), combined renal and gastrointestinal complications (estimated OR, 1.46; 95% CI, 0.58 to 3.69; *P* =  0.42), and length of postoperative hospital stay in days (incidence rate ratio, 1.06; 95% CI, 0.92 to 1.22; *P* =  0.39).

## Discussion

In older adult patients who underwent inpatient non-cardiac surgery, receipt of NSAIDs intraoperatively or during postoperative admission was associated with a 78% increase in odds of a composite of postoperative cardiovascular complications, which did not reach statistical significance. Although the result was of marginal statistical significance (*P* =  0.06), with the overall incidence of cardiovascular complications of 2%, the number needed to harm (NNH) was large at 176. Thus, NSAIDs were not associated with a clinically meaningful increase in the risk of cardiovascular complications.

The effect size of NSAIDs on cardiovascular complications in our study is mainly driven by ibuprofen and ketorolac at relatively low doses (i.e., median doses 2400 mg for ibuprofen and 60 mg for ketorolac), as very few patients received celecoxib or indomethacin. The sub-analyses of patients who received ibuprofen and ketorolac revealed similar effect sizes on the outcome (estimated OR of 1.68 and 1.31, respectively). Among the patients who received ibuprofen, the patients who received a lower dose (≤  2400 mg) had an incidence of cardiovascular complications of 2.68%, and those who received a higher dose (>  2400 mg) had an incidence of 2.37%. Among the patients who received ketorolac, the patients who received a lower dose (≤  60 mg) had an incidence of cardiovascular complications of 1.94%, and those who received a higher dose (>  60 mg) had an incidence of 2.52%. Combining the patients who received ibuprofen and ketorolac, the incidences of cardiovascular complications were 2.3% for the lower dose group (i.e., ibuprofen ≤  2400 mg or ketorolac ≤  60 mg) and 2.4% for the higher dose group (i.e., ibuprofen >  2400 mg or ketorolac >  60 mg), which are almost identical. Therefore, at least in the dose range of ibuprofen and ketorolac given in the current study, no dose–response relationship between NSAID dose and cardiovascular complications was observed, and short-term perioperative use of ibuprofen and ketorolac in older adults is probably safe.

In a meta-analysis of studies of non-surgical patients in which a similar definition of cardiovascular events as our study was employed, the relative risk of cardiovascular events for non-selective NSAIDs was 1.18, and that specific to ibuprofen was 1.17 [13], which is grossly in agreement with those in our study despite the duration of NSAID use was much shorter in our study. Data specific to the association between perioperative use of NSAIDs in older adults and postoperative cardiovascular events are scarce. In the study of Liu et al., where the association between short perioperative use of NSAIDs and postoperative myocardial infarction as defined by troponin I level >  0.1 ng/ml was investigated in older adult patients undergoing total hip or knee replacement, the adjusted relative risk of myocardial infarction was 0.9 for those who received non-selective NSAIDs in comparison to those who did not receive NSAIDs [14]. The study did not investigate other cardiovascular complications, and the non-selective NSAID preparations used were different from our study (i.e., meloxicam and ketorolac) [14], which makes direct comparison to our study difficult.

As the incidences of renal and gastrointestinal complications were low in our study, we analyzed the association between NSAIDs and a combined composite of renal and gastrointestinal complications which resulted in an estimated OR of 1.30. With the overall incidence of the composite of complications of 1.5%, NNH was 157**,** which indicated no clinically meaningful increase in the risk of the composite with the use of NSAIDs. Several studies have investigated the association between postoperative kidney injury and perioperative NSAID use in older adult surgical patients. Heins et al. found no association between postoperative kidney injury as defined by ICD-9 diagnostic codes and the use of various NSAIDs given on the day of surgery in patients ≥  65 years of age who had hip surgery for fracture. Ketorolac (mean dose 42.3 mg) was the most commonly used preparation, and no effect size in the form of adjusted OR or relative risk was given as the incidence of kidney injury was only 0.12% [15]. However, the incidence of acute kidney injury as defined by Kidney Disease Improving Global Outcomes (KDIGO) criteria in the similar population who received ketorolac has been reported to be 2.5% [16], and thus many kidney injury events might have been left undetected in that study. In a study of younger patients undergoing resection of the gastrointestinal tract and hepatobiliary surgery, NSAIDs given during postoperative days 0 to 3 were not associated with acute kidney injury by postoperative day 7 as defined by KDIGO criteria with adjusted OR of 0.8 [17]. The incidence of acute kidney injury was higher in that study than our study, which could be explained by that NSQIP-defined progressive renal insufficiency and acute renal failure require a much larger increase of serum creatinine than acute kidney injury defined by KDIGO criteria.

For gastrointestinal complications, the same study identified that perioperative NSAIDs were not associated with an anastomotic leak in patients who had an anastomosis of the gastrointestinal tract with an adjusted OR of 0.85 [17]. However, the composite of gastrointestinal complications in our study consists of items of gastrointestinal bleeding and ulcers, and we could not identify studies of perioperative NSIAD use that could be directly compared to our study.

There are several limitations inherent to our study. The effect size of the association between perioperative NSAIDs and the outcomes was mainly driven by ibuprofen and ketorolac, and only 11 patients received celecoxib in our study. Therefore, the results of our study are not applicable to Cox-2 selective inhibitors, and also probably not to other non-selective NSAIDs such as diclofenac which has been shown to be associated with a higher risk of cardiovascular events than ibuprofen in non-surgical patients [13]. Despite adjustment for confounding variables by inverse probability of treatment weighting, our study suffers from some degree of residual confounding. For example, as the NSQIP registry lacks detail about baseline medications, we could not include beta-blockers, angiotensin-converting enzyme inhibitors, and antiplatelet or anticoagulant drugs, which might have affected the incidence of cardiovascular, renal, and gastrointestinal bleeding complications. To assess the possible impact of extreme weights, we performed a sensitivity analysis, excluding observations having estimated weights lower than the 1st percentile or larger than the 99th percentile. The sensitivity analysis did not change the point estimates of the effect size of associations between NSAIDs and the outcomes from those in the primary analysis, which indicates results of our analyses were robust. The ICD-9 and ICD-10 diagnosis codes were used to identify the items of gastrointestinal complications. As opposed to items of cardiovascular and renal complications included in the NSQIP registry for which reliability is enhanced by consistent data collection and auditing, some degree of inaccuracy might have been introduced. The incidence of major postoperative complications based on ICD coding has been reported to be similar to that based on a prospective assessment of complications, but ICD coding captured many medical events of limited clinical importance and tends to inflate the overall incidence of complications [18].

## Conclusions

NSAIDs given intraoperatively, or during postoperative admission were not associated with increased incidences of postoperative cardiovascular complications, and renal and gastrointestinal complications within 30 days, or length of postoperative hospital stay in older adults undergoing inpatient non-cardiac surgery.

### Supplementary Information

**Additional file 1: Table S1.** Items of postoperative gastrointestinal complications

## Data Availability

The data that support the findings of this study are available from the corresponding author, RK, upon reasonable request.

## Abbreviations

NSAIDs: Non-steroidal anti-inflammatory drugs
OR: Odds ratio
CI: Confidence interval
Cox: Cyclooxygenase
ACS-NSQIP: American College of Surgeons-National Surgical Quality Improvement Program
STROBE: Strengthening the Reporting of Observational Studies in Epidemiology
CPR: Cardiopulmonary resuscitation
ICD: International Classification of Diseases
ASMD: Absolute standardized mean difference
NNH: Number needed to harm
KDIGO: Kidney Disease Improving Global Outcomes
