# Supplementary material for: Association between perioperative non-steroidal anti-inflammatory drug use and cardiovascular complications after non-cardiac surgery in older adult patients
Source: JA Clin Rep. 2024 Apr 30;10:29. doi: 10.1186/s40981-024-00712-5 (PMC11061052; doi:10.1186/s40981-024-00712-5)
Supplement: Supplementary file 1 — Additional file 1: Table S1. Items of postoperative gastrointestinal complications [file 40981_2024_712_MOESM1_ESM.docx]

**Supplemental Table 1.** Items of postoperative gastrointestinal complications.

| **Items of complications** | **ICD-9 code** | **ICD-10 code** |
| --- | --- | --- |
| Acute gastric ulcer without hemorrhage or perforation | 531.3 | K25.3 |
| Gastric ulcer, unspecified as acute or chronic, without hemorrhage or perforation | 531.9 | K25.9 |
| Acute duodenal ulcer without hemorrhage or perforation | 532.3 | K26.3 |
| Acute peptic ulcer, site unspecified, without hemorrhage | 533.3 | K27.3 |
| Acute gastritis without bleeding | 535 | K29.00 |
| Other gastritis without bleeding | 535.4 | K29.60 |
| Other gastritis with bleeding | N/A | K29.61 |
| Duodenitis without bleeding | 535.6 | K29.80 |
| Duodenitis with bleeding | N/A | K29.81 |
| Hemorrhage of anus and rectum | 569.3 | K62.5 |
| Hematemesis | 578 | K92.0 |
| Melena. Blood in stool | 578.1 | K92.1 |
| Gastrointestinal hemorrhage, unspecified | 578.9 | K92.2 |

Abbreviations: ICD = International Classification of Diseases, N/A = not applicable
